# Acousto-optically driven lensless single-shot ultrafast optical imaging

**DOI:** 10.1038/s41377-022-00759-y

**Published:** 2022-03-23

**Authors:** Mohamed Touil, Saïd Idlahcen, Rezki Becheker, Denis Lebrun, Claude Rozé, Ammar Hideur, Thomas Godin

**Affiliations:** CORIA, CNRS UMR6614—Université de Rouen Normandie—INSA Rouen, 76800 Saint Etienne du Rouvray, France

**Keywords:** Imaging and sensing, Optical metrology

## Abstract

Driven by many applications in a wide span of scientific fields, a myriad of advanced ultrafast imaging techniques have emerged in the last decade, featuring record-high imaging speeds above a trillion-frame-per-second with long sequence depths. Although bringing remarkable insights into various ultrafast phenomena, their application out of a laboratory environment is however limited in most cases, either by the cost, complexity of the operation or by heavy data processing. We then report a versatile single-shot imaging technique combining sequentially timed all-optical mapping photography (STAMP) with acousto-optics programmable dispersive filtering (AOPDF) and digital in-line holography (DIH). On the one hand, a high degree of simplicity is reached through the AOPDF, which enables full control over the acquisition parameters via an electrically driven phase and amplitude spectro-temporal tailoring of the imaging pulses. Here, contrary to most single-shot techniques, the frame rate, exposure time, and frame intensities can be independently adjusted in a wide range of pulse durations and chirp values without resorting to complex shaping stages, making the system remarkably agile and user-friendly. On the other hand, the use of DIH, which does not require any reference beam, allows to achieve an even higher technical simplicity by allowing its lensless operation but also for reconstructing the object on a wide depth of field, contrary to classical techniques that only provide images in a single plane. The imaging speed of the system as well as its flexibility are demonstrated by visualizing ultrashort events on both the picosecond and nanosecond timescales. The virtues and limitations as well as the potential improvements of this on-demand ultrafast imaging method are critically discussed.

## Abstract (web summary)

We demonstrate a versatile and user-friendly ultrafast single-shot imaging technique based on a simple and flexible acousto-optics spectro-temporal shaping stage combined with digital in-line holography.

## Graphical abstract

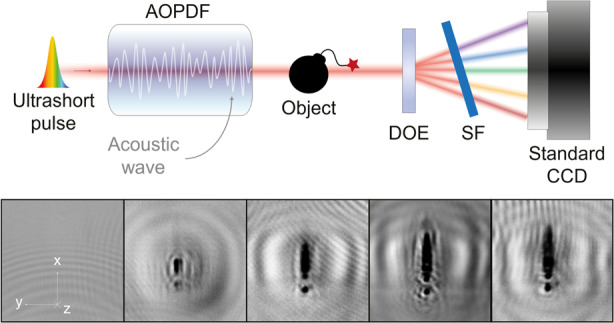

## Introduction

Capturing the transient dynamics of ultrashort events focuses the attention of the scientific community for decades in biomedical science, chemistry, and physics^1–4^, and ultrafast imaging is now routinely used for research and industrial applications. The need for high temporal and spatial resolutions has then remarkably fueled unprecedented advances in ultrafast optical imaging, and sub-picosecond and sub-nanometer resolutions are now accessible to various technologies. Transient phenomena are traditionally captured using pump-probe methods, which are however intrinsically limited to highly repeatable experimental conditions^5–7^. Some techniques, such as time-stretch imaging^8^, leverage their high throughput capabilities for recording high-speed processes on long—microsecond timescales with frame rates in the order of a hundred million of frames-per-second (fps)^9,10^. Nevertheless, such frame rates are not sufficient to resolve many ultrafast events and single-shot techniques with frame intervals in the picosecond range—or less—are required for imaging e.g., laser-induced phenomena^11,12^ or light propagation itself^13,14^. Single-shot methods can be separated between passive and active detection and further between direct imaging and reconstruction imaging^6,15^, each family having its strengths and limitations. Passive detection refers to the use of custom-designed pulse trains to probe ultrashort events while active detection relies on specific ultrafast detection schemes. On the one hand, the best performances to date in terms of imaging speed and sequence depth are undoubtedly obtained with passive detection and image reconstruction using advanced algorithms such as compressed sensing. Among those computational imaging methods, compressed ultrafast photography (CUP)^16^ and its noteworthy upgradings and variants^11,13,17–20^ then manage to reach up to 70 trillion Hz frame rates^21^ and theoretically up to more than 180 Tfps^22^, few hundreds of femtosecond frame intervals, and potentially sequences with hundreds of frames, in single camera exposure. Albeit being the spearhead of ultrafast imaging techniques and particularly relevant in a laboratory environment, these techniques could suffer from their complex reconstruction scheme hindering the real-time operation and from their low flexibility. On the other hand, active imaging techniques using spatially, temporally, and/or spectrally tailored ultrafast pulse trains have enabled ultra-high frame rates with simpler experimental setups and without the need of complex computational algorithms^14,23–25^ but, in most cases, to the detriment of sequence depth and without the possibility to record the spectrum (e.g., fluorescence) emitted from the dynamic scene. Among them, sequentially timed all-optical mapping photography (STAMP)^26^) and more specifically its spectrally filtered variant (SF-STAMP)^27,28^ offer trillion-fps rates with the real-time operation but suffer, as most of the above-mentioned techniques, from the absence of adaptability of its main parameters—namely, frame rate and exposure time—that cannot be independently adjusted.

In order to provide a genuinely flexible and user-friendly system that can provide ultra-high frame rates while keeping a high simplicity of use, we introduce here an agile technique combining spectrally filtered STAMP with compact acousto-optics-based electronically controllable phase and amplitude filters and digital in-line holography. On the one hand, the acousto-optic programmable dispersive filter (AOPDF) strikingly allows to perform the spectro-temporal phase and amplitude shaping stages at once and in a very simple manner, then circumventing the complex and bulky systems that are usually sequentially used. AOPDFs have never been used for single-shot imaging and have originally been designed to compensate for the group delay dispersion in ultrafast laser systems^29^, carrier-envelope phase stabilization^30,31^, or pulse shaping in various applications^32–34^. AOPDF have also been used as ultrafast delay lines in terahertz spectroscopy^35^ and pump-probe spectroscopy and imaging^36^. Their ability to fully tailor the pulse phase and amplitude in a simple way through the interaction of the light pulse with an acoustic wave also enabled the synchronization and chirp control of fs pulses, e.g., in stimulated Raman spectroscopy^37,38^. On the other hand, the use of digital in-line holography (DIH) can also bring the system to an even higher degree of simplicity through its lensless operation but also by allowing the reconstruction and localization of the object on a wide depth of field, contrary to classical techniques that only provide images in a single plane. Digital holography has previously been used for time-resolved imaging^23,25,39,40^ but DIH is advantageous as it does not require a reference arm nor complex reconstruction algorithms, then enabling real-time operation. In addition, contrary to standard digital holography, DIH stands out as it allows fast object tracking along a single line-of-sight^41^ but has never been used within a single-shot Tfps imaging setup. We then propose here to leverage the AOPDF pulse-shaping capabilities and the DIH simplicity in a flexible SF-STAMP ultrafast imaging scheme, and demonstrate the independent control of frame rate, exposure time, and frame intensities as well as the ultrafast imaging of ultrashort light-induced phenomena on different timescales. The potential refinements and limitations of the method in a real-world environment are eventually discussed.

## Results

### AOPDF-based sequentially timed all-optical mapping photography: principle and parameters control

In spectrally filtered sequentially timed all-optical mapping photography experiments, broadband chirped pulses are used to illuminate the object under study, each frequency then capturing snapshots of the dynamic scene at different instants^26^. The different wavelengths are subsequently spatially separated using the combination of a diffractive optical element (DOE) and a tilted spectral filter (SF) and the generated sub-pulses, corresponding to the consecutive frames, are recorded using a standard camera^27^. As the snapshots are obtained through the spatial separation of specifically selected spectral bands from the main spectrum of the laser pulse, the exposure time of the technique is then defined by the spectral width of the extracted sub-pulses, and by the initial chirp of the broadband input pulse. The time between frames is also determined by the initial chirp along with the spectral gap between consecutive sub-pulses. For standard SF-STAMP, the DOE divides the input pulse into several replicas propagating in different directions while, for each replica, the angle of incidence on the spectral filter fixes the transmitted central wavelength. The limited bandwidth of the input ultrashort pulse intrinsically limits the freedom in the azimuthal and radial angles of the spectral filter, and thereby hinders the acquisition of equally spaced non-overlapping (in the spectral domain) sub-pulses. Prior to the illumination of the object, pulses are then temporally stretched and spectrally shaped in order to achieve the desired frame rates and exposure times. The pulse stretching step usually consists in propagating in glass rods or dispersive elements such as prisms while the spectral broadening and/or shaping is obtained via nonlinear propagation (SPM) in optical fibers or using more complex setups based on 4-f lines with spatial light modulators^26,28^. Such pulse-shaping stages, in addition to being bulky, exhibit very low flexibility as the frame rate and exposure time are fixed and cannot be independently controlled by the end-user.

**Fig. 1:**
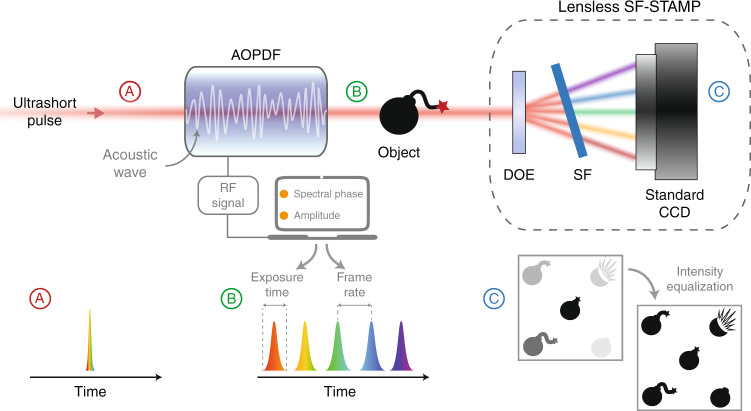
Principle of operation. The acousto-optics programmable dispersive filter (AOPDF) tailors the pulse shape in both the spectral and temporal domains via its interaction with an electrically driven acoustic wave, then enabling the full and independent control over the exposure time and frame rate in the subsequent SF-STAMP detection scheme, comprising a diffractive optical element (DOE), a tilted spectral filter (SF) and a standard camera.

Here, we then circumvent these steps using a single acousto-optics programmable dispersive filter (AOPDF) for both temporal and spectral shaping and then for the full control of the illumination conditions, as shown in Fig. 1. It consists of a centimeter-long birefringent crystal where the optical pulse interacts with a radio-frequency-driven acoustic wave, enabling the simultaneous and independent tailoring of its spectral phase and amplitude. The wavelength-dependent group delay and amplitude are then controlled within a compact, turn-key device, allowing to precisely tune both the exposure time and frame rate in real time, but also to guarantee that the diffracted replicas exhibit comparable intensities and thereby to maximize the camera dynamics (lower panel in Fig. 1). Therefore, the SF-STAMP system is simplified and reduced to only three main components: a laser source that generates laser pulses with a spectral width of only 10 nm, the AOPDF, and the DOE/SF pair. Among these components, the only moving part is the SF. This minimizes the tedious task of optical alignment. As a result, the proposed system can be accessible to non-specialists for routine use.

**Fig. 2:**
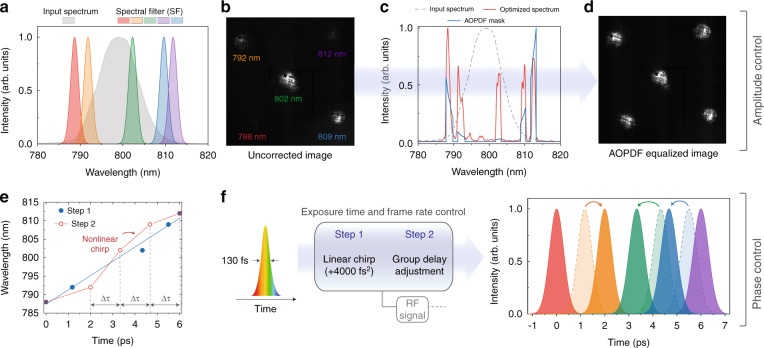
AOPDF-based amplitude and phase control. **a** Input laser spectrum compared with the normalized transmission bands of the STAMP spectral filter (SF). **b** The convolution of the input spectrum with the SF leads to an inhomogeneous sub-pulse intensity distribution on the CCD camera and is associated with poor intensity dynamics. **c** Amplitude mask applied on the AOPDF (blue) and resulting optimized spectrum (red) compared with the input spectrum (dotted gray). **d** Resulting image with equalized intensities and optimized dynamics. These are raw images without any post-processing. **e, f** Spectro-temporal distributions of the illuminating pulses. Exposure time and frame rate can be independently controlled by adding a linear chirp and adjusting the group delay of each spectral component, respectively. The arrows correspond to the group delay effect on the sub-pulses.

We highlight the flexibility of our concept with the example of amplitude and phase shaping shown in Fig. 2. The input laser pulse spectrum is compared with the optimal transfer functions of the SF for each of the sub-pulses (five replicas in our case) in Fig. 2a, corresponding to an optimized set of azimuthal and radial angles. Although the five transfer functions fall within the spectral range of the input pulse spectrum, there is considerable overlap—spectral and temporal- between some of them and only three snapshots can therefore be acquired. The other major drawback is their low overlap with the input spectrum and then the huge contrast between the central image at 802 nm and the other ones, resulting in very poor intensity dynamics, as shown in Fig. 2b. Considering a linearly chirped input pulse, this also results in a non-constant time between frames.

In order to overcome these limitations, we then performed an AOPDF-based amplitude and phase control. In the AOPDF crystal, the laser pulse interacts with a control field in the acoustic domain through a frequency mixing process where the energy is quasi-conserved. When the phase-matching conditions between the acoustic and optical waves are reached, the optical field is diffracted from the ordinary to the extraordinary axis of the crystal at the location of interaction and the intensity of the diffracted light will be proportional to that of the corresponding phase-matched acoustic wave. By properly tailoring the control field, the output spectrum can thereby be precisely shaped together with the location in the crystal where each component is diffracted. Therefore, a predetermined delay is created between the selected spectral bands, which can be fully controlled until a maximum value defined by the length of the crystal and the refractive index difference between its axes. Figure 2c shows the amplitude mask designed to generate the desired control acoustic field and the subsequently shaped output spectrum featuring quasi-equalized spectral bands. This allows to exploit the full dynamic range of the sensor (see Fig. 2d) while preventing any spectral or temporal overlap between the five sub-pulses.

In order to increase the diffraction efficiency of the frequency mixing process, the designed acoustic wave is set to cover most of the crystal length when interacting with the unchirped input pulse. This allows to efficiently obtain a linearly chirped output pulse with an equivalent accumulated dispersion D = 0.269 ps/nm (blue line in Fig. 2e). Based on this accumulated dispersion and on the spectral width of each sub-pulse, the exposure time (i.e., the pulse width of the sub-pulses) is estimated to an average value of 723 fs. At this stage, the time between frames corresponds to the separation between the consecutive sub-pulses. In order to even this time between frames, the position at which each of the five sub-pulses is diffracted within the crystal is adjusted via an additional delay (positive or negative) resulting in the temporal shift of the selected sub-pulses (red curve in Fig. 2e and Fig. 2f). We then obtain a single-shot ultrafast imaging system with an average exposure time of 723 fs and time between frames of 2 ps between the two first images and 1.33 ps between the next ones. We then overcome one of the main limitations of the STAMP technique as the time between the five frames can be independently equalized while the exposure time is left unchanged. This remarkable agility is obtained only through a single user-friendly electronically controlled pulse-shaping system and without resorting to any additional spectral broadening and temporal stretching stages.

### Ultrafast imaging of an optical Kerr gate

The temporal capabilities of ultrafast systems are usually validated by imaging femtosecond- or picosecond-scale light-induced phenomena such as the propagation of a pump pulse through a Kerr medium. In order to demonstrate that our flexible technique is on par with state-of-the-art active imaging methods, we then used our system to capture the opening and closing of a CS_2_-based optical Kerr gate (OKG). The principle of an OKG relies on placing a transparent medium with a high nonlinear refractive index (liquid CS_2_ here) between two crossed polarizers along the optical axis of an image beam. An intense ultrashort pump pulse is then used to induce a local birefringence which results in a change of the polarization state of the image beam from linear into elliptical, therefore allowing the partial transmission of the image beam through the second polarizer. The gate time response depends on the pump pulse duration in addition to the relaxation time of the nonlinear medium. OKGs are thereby simple and efficient time gates and are routinely used e.g., for capturing ballistic photons (and rejecting multi-scattered photons) in ultrafast imaging experiments^42^.

**Fig. 3:**
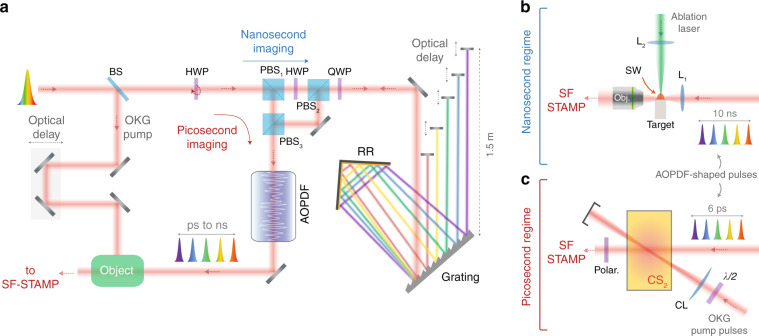
AOPDF-based ultrafast imaging system. **a** Experimental setup. A half-wave plate allows to either operate the system in the picosecond or nanosecond regime. The latter is obtained using a grating-based stretcher with an additional adjustable delay between the sub-pulses prior to the AOPDF spectro-temporal shaping. **b** Experimental configuration used to generate and capture laser-induced shock waves (SW) on the nanosecond regime. SW are generated by focusing intense visible pulses onto solid targets. **c** Experimental configuration used for the picosecond-scale imaging of an optical Kerr gate (OKG) in a CS_2_ cell. An optical delay line is used to synchronize the pump pulses with the imaging pulses in the CS_2_ cell and to precisely capture the Kerr gate dynamics.

**Fig. 4:**
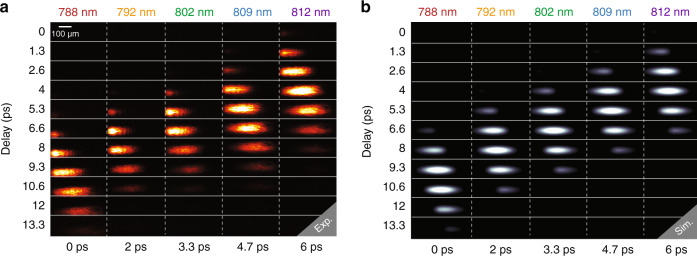
Imaging of an optical Kerr gate (OKG) in the picosecond regime. **a** Vertically stacked images of the transmitted pulses while varying the optical delay. The opening and closing of the OKG are fully acquired for a delay of 6.6 ps. **b** Numerical simulation (see "Methods") of the OKG imaging process.

We then used our AOPDF-based system to capture the whole OKG process using the setup depicted in Fig. 3a, c. A linearly polarized fs source is used for both the imaging via the pulse-shaping stage and the CS_2_ pumping via an optical delay line (ODL). On the pump arm, the ODL allows to synchronize the pump and imaging pulses and a cylindrical lens is used in order to increase the intensity of the pump within the CS_2_. For each optical delay (step of 400 µm), the imaging system then captures five single-shot images of the OKG, as shown in Fig. 4a where the images are displayed in a matrix in which the rows represent the single-shot images while the columns represent the group optical delay. For a delay of 6.6 ps, the five frames capture the whole pump-induced process, from the opening to the closing of the time-gate. A horizontal spatial displacement of the intensity can also be noticed, which corresponds to the propagation of light within the CS_2_. This displacement would not be noticed on the images illuminated by the fourth and fifth sub-pulses without the adjustment of the time between frames considering the limited spatial resolution. On the one hand, the central wavelengths of these sub-pulses are relatively close and lead to a temporal separation of only 750 fs when group delay adjustment is not performed. On the other hand, the exposure time is fixed to a value of 723 fs. This leads to a significant temporal overlap and the two sub-pulses capture the optical Kerr gate at very close instants, and a significant difference between the two acquired images is hardly observable. This highlights the importance of the time between frames adjustment using the AOPDF. In order to further confirm the ability of our system to provide accurate measurements, the propagation of the pump and imaging pulses within the CS_2_ cell have been simulated using a validated standard model^42^. The latter allows to predict the transmitted light from the second polarizer as a function of the pump and image pulses parameters by modeling the CS_2_ response using a single relaxation process (see “Methods” section). The simulated snapshots for each spectral component, in full agreement with the experimental measurements, are shown in Fig. 4b.

### Extending the frame interval to the nanosecond

As a lot of transient or non-repeatable phenomena (e.g., in light-matter interactions) also occur on the nanosecond time scale, and to illustrate the flexibility of our AOPDF-based imaging technique, we demonstrate its extension to nanosecond frame intervals. The extension of the sub-ps initial temporal resolution of SF-STAMP to the ns range has already been demonstrated e.g., using free-space angular-chirp-enhanced delay (FACED)^9,43^, but to the detriment of simplicity as the frame rate and exposure time are intrinsically related in such techniques. Here we propose to extend the frame interval through the increase of the group delay between the five sub-pulses while keeping their respective pulse width close to the Fourier limit, thereby minimizing the ratio between exposure time and time between frames.

Here, the accessible time scale is intrinsically fixed by the length of the acousto-optic crystal (a few cm) and thereby limited to a few picoseconds. Therefore, we added the extension shown in Fig. 3a to the imaging system for generating a controlled group delay with an extended and adjustable temporal window. This extension is set prior to the AOPDF as its crystal has a damage threshold of ~30 µJ and, because of the efficiency of the shaping process as well as the amplitude equalization, only a fraction of the input pulse is recuperated at the output. This fraction would then undergo the extra losses of the nanosecond extension and this would result in insufficient sub-pulses intensities to properly image the object of interest. Consequently, placing the nanosecond extension setup prior to the AOPDF allows to compensate for its inherent losses by increasing the pulse energy at the laser level and therefore ensures sufficient pulse energy at the output of the AOPDF. In the implementation of the nanosecond extension setup, we used a modified single grating pulse compressor comprising a diffraction grating, a roof mirror retroreflector for horizontal displacement and five moving mirrors for each of the five sub-pulses. The single grating compressor configuration is chosen in order to keep the system as compact as possible.

**Fig. 5:**
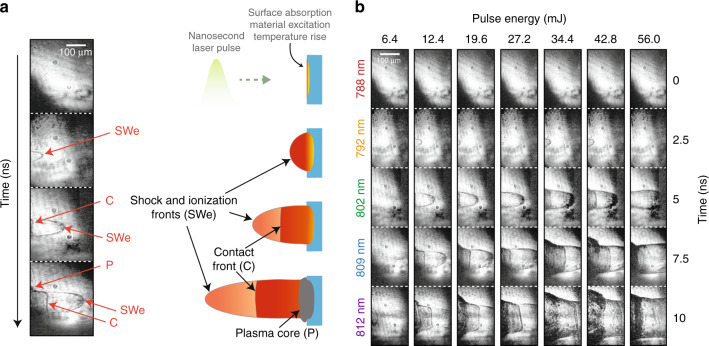
Capture of laser-induced ablation dynamics in the nanosecond regime. **a** Birth and evolution of laser-induced gas dynamics captured using our system and illustration of the physical phenomena involved. The shock and ionization front (external shock wave, SWe), as well as the contact front evolution, are clearly observed. **b** Monitoring of gas dynamics during laser ablation on glass while varying the pump pulse energy.

As the input laser beam is linearly polarized, a half-wave plate is used prior to a polarizing beam splitter (PBS) to inject the initial pulse either directly in the AOPDF for ps-scale imaging or towards the grating-based extension to reach longer timescales. The orientation of the PBS is chosen so that the polarization of the exiting beam toward the AOPDF is appropriate for the frequency mixing process. A quarter-wave plate is also used to ensure that the back-propagating beam is efficiently coupled into the AOPDF after the phase modulation of the input beam. The modified single grating pulse compressor induces a negative group velocity dispersion (GVD) as an ordinary single grating compressor, and the distance between the grating and the roof mirror determines the induced GVD in addition to defining the horizontal spacing between the sub-pulses after the second reflection from the grating. Using five spatially separated moving mirrors allows to generate a group delay between each spectral band eventually resulting in a predetermined time difference between the five imaging sub-pulses at the output of the AOPDF. In this process, the AOPDF plays the role of refining the spectrum by eliminating any existing spectral overlap between the sub-pulses. It also allows to equalizes their amplitude as described in the previous section. In addition, the positive GVD induced by the AOPDF partially compensates for the negative GVD experienced in the compressor, then minimizing the average pulse duration of the sub-pulses. As a result, this simple modified setup allows to extend the time scale of the imaging system to the nanosecond regime while keeping the exposure time inferior to one picosecond, a feature that allows the imaging system to obtain sharp images and avoid the blur effect. In order to validate its nanosecond-scale imaging capabilities, we then applied the modified technique to the tracking of shock waves as well as other laser-induced discontinuities in the ablation process. Laser ablation is performed by focusing high-energy 532 nm nanosecond pulses on glass samples using a lens with f = 12 mm, as shown in Fig. 3b. Although the window of observation is enlarged over 1600 times, the frame rate is still too high to capture shock waves in motion. For this reason, we resorted to the reduction of the field of view in order to be able to observe the dynamics of the laser ablation process. This way, the evolution of the discontinuities is captured using the standard SF-STAMP system as shown in Fig. 5 without the need for any post-processing. The high ratio between exposure time and time between frames along with intensity equalization allowed for the precise visualization of the gas dynamics in the laser ablation process. Moreover, the displacements of the shock waves as well as the different discontinuities can be precisely and easily inferred. In Fig. 5a, we can indeed clearly observe the birth and development of the discontinuities with particular dynamics. The latter can be explained as follows. When the intense laser pulse illuminates the ablation area, it causes its liquefaction and evaporation. The high velocity of the evaporated material into the ambient air leads to its compression in a thin shell. Therefore, a strong nearly transparent external shock front is formed. This front is almost attached to an ionization front that consists of shocked and ionized air. Afterwards, the ablated material produces a contact front with a planar shape confining vapor and plasma from the target material, and the composition of the confined gas explains the contrast of this region with respect to the air confined by the external shock wave. The possible existence of plasma ions and free electrons leads to a high absorption of light and, in addition, the high-density vapor can contribute to this contrast. Finally, an even darker region with a planar shape is created above the ablated area defining the plasma core.

In addition, in order to further illustrate the operation of the technique in the ns regime, we performed single-shot acquisitions while varying the pulse energy as shown in Fig. 5b. In this experiment, the pump laser was focused in the air at a distance of 60 µm from the glass target. With the pulse energy of 6.4 mJ, the ionization threshold was just reached and a relatively slow-moving shock wave is created. As the pulse energy is increased, the birth of the discontinuities occurs faster after the illumination of the ablated area. The propagated distance of the discontinuities increases with the pulse energy. However, their shape remains relatively the same. With the pulse energy of 12.4 mJ, the five snapshots were sufficient to capture the creation and movement of the shock, ionization, and contact fronts as well as the plasma core. At higher pulse energies, we noticed an increase in the contrast of the plasma core region. The tracking of these phenomena then demonstrates the versatility of the system, whether operating in the picosecond or nanosecond regime.

### Lensless imaging based on digital in-line holography

**Fig. 6:**
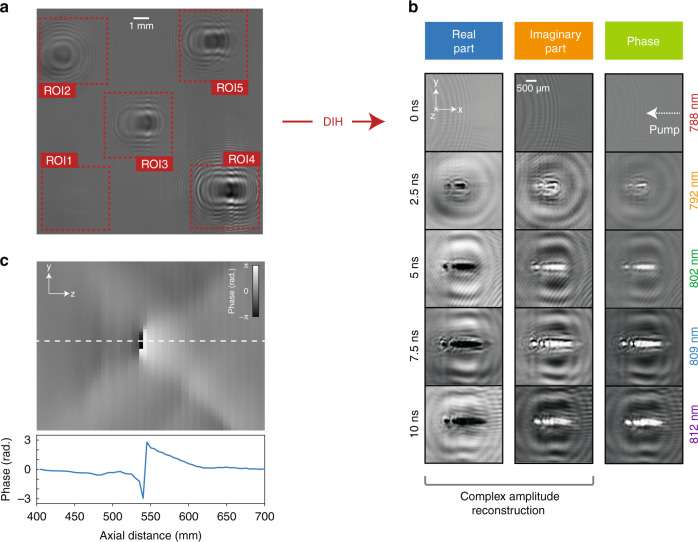
Image reconstruction using digital in-line holography (DIH). The concept of lensless AOPDF-based SF-STAMP based on DIH is demonstrated by imaging laser-induced air breakdown on the ns scale. **a** Hologram normalized by background subtraction. **b** Complex amplitude and phase reconstruction using DIH (based on the hologram of a). The image is reconstructed at an axial distance z_*r*_ = 550 mm and the asymmetrical plasma expansion is clearly seen. **c** Phase map along the longitudinal axis obtained by reconstructing the complex amplitude and calculating the phase at different longitudinal coordinates. The selected zone of interest is the leftmost spot in the air breakdown pattern in **b**. The phase variation along the optical axis (blue solid line) is a striking signature of the actual location of the intensity and phase object.

In our system, as in most the single-shot imaging techniques, the dynamic scene is captured through a combination of optical elements such as lenses, e.g., to access a given field of view. Such lenses however require a careful alignment from the end-user and ultrafast imaging techniques could gain in simplicity if this step was removed. With this in mind, the use of digital holography is then a natural solution. In a different scope, digital holography has previously been directly used as a space-division light-in-flight recording technique^23^. This technique exhibited Tfps frame rates, but was somehow limited in terms of spatial resolution and required a reference pulse. Here we then propose to combine the AOPDF-based SF-STAMP technique with digital in-line holography (DIH) as it requires neither any additional optical component nor a reference arm. It is worth mentioning that DIH is indeed very simple, whether from the experimental point of view or regarding the reconstruction algorithms. The image reconstruction using DIH for a specific distance on the optical axis can be performed in real time without requiring advanced computational means, as it is only based on two Fourier transforms and one inverse Fourier transform. In addition, DIH brings two remarkable features: (i) images can be reconstructed in many planes along the *z*-axis, (ii) each image can be independently reconstructed at a different wavelength, which is particularly relevant here as each sub-pulse has a different wavelength in the 788–812 nm range. Then, the use of DIH actually allows to simplify the system even further using approved digital in-line holography algorithms. All the lenses were thereby removed from the setup and holograms, such as the one shown in Fig. 6a, were recorded on the CCD sensor. Note that the hologram in Fig. 6a was simply normalized by the background intensity distribution previously recorded without the plasma excitation beam. From this hologram, the complex amplitude can be reconstructed in any plane along the optical axis using an adapted model (see “Methods”) as shown in Fig. 6b, where the real and imaginary parts of the amplitude are displayed.

In this experiment, ns pulses from a frequency-doubled Nd-YAG laser were focused (using a lens with f = 20 cm) to generate laser-induced air breakdown, which manifests as the specific patterns shown in Fig. 6b. This phenomenon can be explained as follows. When the pulses are focused within a small volume, it initiates laser-accelerated electrons through a multiphoton ionization process, leading to successive cascaded ionizations of the medium by inelastic collisions. This eventually results in the formation of a plasma expanding asymmetrically toward the pump laser^44^. This type of pattern has been observed using classical burst imaging techniques in the ps regime^27^, which only allows to visualize one state of this process (corresponding to the second snapshot in Fig. 6b). Here, the birth and growth of this pattern can clearly be seen in the consecutive snapshots in the reconstructed images obtained through the lensless AOPDF-based technique. From these real and imaginary part images, the phase can also easily be calculated using *ϕ*(*x*,*y*) = *arctan*(*Im*(*x*,*y*)*/Re*(*x*,*y*)), as shown in 6b. Remarkably, calculating the phase is an efficient way to precisely determine the axial coordinate *z* at which the object of interest (here, the highest electron density region) is located using the following procedure. From the complex amplitude images, the phase images are calculated at every axial coordinate and stacked horizontally in order to plot an orthogonal view of the phase map such as the one shown in Fig. 6c. The phase profile along the optical axis (red solid line) exhibits a typical shape that has been observed previously^45^, which is a clear signature of the object position and then gives a simple and efficient tool for accurate focusing on the best-reconstructed image.

## Discussion

### Limitations and potential enhancements

The SF-STAMP technique on which relies our system has proven efficient for capturing ultrafast events in a simple and user-friendly manner. However, its sequence depth (i.e., the number of images acquired in a single shot) is intrinsically limited by two factors. On the one hand, as it exploits bandwidth-limited laser pulses together with a spectrum sampling process, having a larger number of images would necessitate the filtering and separation of very narrow adjacent spectral bands, which is feasible but could require a more complex setup. In that case, specific attention should be paid to the potential temporal overlap between the extracted spectral bands. On the other hand, the number of images is also limited by the spatial separation of the sub-pulses (through the DOE and tilted spectral filter) prior to the acquisition of the CCD sensor. As a single CCD sensor is used, the larger the number of images, the lower the number of pixels per image and, consequently, a compromise between the sequencing depth and the spatial resolution has to be found. With a view to provide a compact and simple system, the above-mentioned drawbacks could be easily suppressed using several technical solutions leveraging the AOPDF spectro-temporal tailoring capabilities, as it allows the generation of adjacent non-overlapping narrow (sub-nm) spectral bands. It would then be possible to use a broadband fiber laser as the illumination source to increase the number of shaped sub-pulses and a hyperspectral camera to replace the DOE, spectral filter and CCD sensor at once. The number of images would therefore be determined by the number of spectral bands and be independent of the spatial resolution. This would also allow to eliminate the path difference between the sub-pulses due to the DOE that results in a shift along the *z*-axis when reconstructing the images using DIH.

In this work, the main limitation of the AOPDF system is the optical delay that can be generated between two specific wavelengths in the crystal, as it leads the imaging system to exhibit a fixed maximum temporal window of 8.5 ps regardless of the number of images. This maximum group delay also influences the possible number of sub-pulses that can be created as the imaging system imposes a minimal temporal overlap between the generated sub-pulses in order to capture the image of the object at different instants. Therefore, the spectral width of each of the sub-pulses needs to be sufficient to minimize the exposure time (frequency-time duality) and to maximize the number of possible sub-pulses. It is worth noting that longer crystals than the one used in this feasibility demonstration could be used. This would enable larger temporal windows (through larger GDD values) in the picosecond regime. Amplitude wise, the AOPDF is limited by its shaping resolution. This sets a limit to the number of non-overlapping spectral bands to which the optical spectrum can be divided, and consequently to the number of possible sub-pulses. In the nanosecond regime, the temporal window is enlarged using a modified single grating compressor which is however bulky and necessitates a careful alignment. However, the aim of implementing such a system is to induce a significant group delay between different spectral ranges using ordinary optical elements prior to the acousto-optic crystal. This way, it is possible to investigate the shaping of a 10 ns pulse using the AOPDF, a system that is usually devoted to much shorter pulses. It is well known that there is a large difference in the velocity of propagation between the light pulse and the acoustic wave within the crystal and the acoustic wave is then considered to be stationary compared to the optical wave. This assumption is valid for optical pulses up to a certain duration. Using the nanosecond extension setup, we could then demonstrate that pulse shaping using the AOPDF is still functional for a pulse width of 10 ns. Encouraged by this demonstration and in the aim of building a compact alignment-free system, the generation of the additional delay between the pulses could be performed using custom-made fiber Bragg gratings (FBG) adapted to the desired imaging temporal window. Regarding holography, it is clear that a rigorous phase map over the whole field of view cannot be estimated—as in off-axis holography—without a perfectly known ref. ^46^. It is also worth noting that the phase distortions in an in-line configuration could be reduced by a phase conjugation technique as shown in ref. ^41^. However, these two potential refinements would entail a much more complex setup, which is out the scope of this study aiming for a user-friendly technique. Here, as far as a small volume is studied around the considered object of interest, in-line holography is completely satisfactory provided that the local wavefront variations remain acceptable for extracting an exploitable phase signature, as shown in Fig. 6.

### Summary

By leveraging the AOPDF’s pulse-shaping capabilities within a burst imaging scheme together with the use of digital in-line holography, our single-shot technique stands out of the current state-of-the-art by its simplicity of use as its main features are easily controllable and adjustable by the end-user. The outcomes of this feasibility demonstration are manifold and could help to bring such ultrafast techniques out of a laboratory environment. (i) The AOPDF acts as an “all-in-one” compact and flexible device that can tailor the phase and/or the amplitude of the input signal on a wide range of pulse duration and chirp values. Unlike most of the existing techniques, the frame rate and exposure time can then be independently adjusted without resorting to complex or bulky spectro-temporal shaping stages. (ii) The system reaches an even higher degree of simplicity by efficiently using digital in-line holography to reconstruct the object on a wide depth of field, a technique that requires neither a reference arm nor complex reconstruction algorithms. In addition, we confirmed that the axial phase profile of the reconstructed images is a striking signature of the object position on the longitudinal axis. (iii) The technique can be easily switched between different timescales as demonstrated here by either recording the dynamics of an optical Kerr gate in the ps regime or the detailed evolution of laser-induced ablation dynamics in the ns regime. Extra refinements could now be added at different levels on the technique (shaping stage, sequence depth, detector) in order to provide a fully integrated alignment-free system for real-world applications requiring Tfps imaging without using any advanced computational imaging method.

## Methods

### AOPDF-based SF-STAMP

In the experimental setup shown in Fig. 3a, we used a chirped-pulse amplifier (Coherent, Inc.) to generate linearly polarized 100 fs pulses centered at 800 nm with a 10 nm bandwidth (FWHM) and a repetition rate of 1 kHz. The beam has a diameter of 3 mm and a maximum average power of 30 mW. A half-wave plate is rotated to operate the imaging system either in the picosecond or the nanosecond regime. This is achieved using a free-space optical circulator composed of three polarizing beam splitters (PBS). When the HWP is rotated so that the laser beam is vertically polarized, the beam exits PBS_1_ toward PBS_3_ to be directly injected into the AOPDF, thus operating the imaging system in the picosecond regime. In this case, the AOPDF creates five pulses with equal intensities, an equal time delay between them and with specifically selected central wavelengths and spectral widths. It is worth mentioning that the vertical polarization is orthogonal to the diffraction plane of the acousto-optic crystal. When the HWP is rotated so that the beam is horizontally polarized, the beam exits PBS_1_ toward PBS_2_ and then propagates through the nanosecond extension. The quarter-wave plate ensures that the returning beam is vertically polarized and exits PBS2 toward PBS3 to be injected into the AOPDF. The nanosecond stage consists of a modified single grating compressor where the laser beam is diffracted by a 600 lines/mm grating set at an angle of 45° with respect to the incoming beam’s optical axis. The first-order diffracted beam is sent back to the grating by a roof mirror reflector set at 300 mm from the grating, thus providing a GVD of −1.28 ps/nm. A collimated spectral line is then created by the grating and sent towards five mirrors set 375 mm apart, resulting in a total distance of 1.5 m between the closest and farthest mirrors with respect to the gratings. As a result, five selected spectral bands are then reflected back to the grating. The first mirror reflects the spectral band up to 790 nm while the rest is left to propagate toward the remaining mirrors. In ascending order, these mirrors reflect the spectral bands up to 800, 807, and 811 nm, and the last mirror reflects the remaining part of the spectrum. A group delay of 2.5 ns between the sub-pulses is then obtained prior to injection in the AOPDF. Here, the spectral widths and central wavelengths of the five sub-pulses can be finely tuned in order to avoid any spectral overlap on the interference filter and their respective intensities can also be equalized. Finally, the GVD induced by the modified single grating compressor can be partially compensated in order to compress the five pulses closer to their Fourier limits.

Before the AOPDF, the beam diameter was reduced to 1.6 mm with a divergence inferior to 0.04° in order to maximize both the resolution and efficiency of the pulse-shaping system. The AOPDF (Dazzler, Fastlite) consists of a 25 mm long TeO_2_ birefringent acousto-optic crystal for spectro-temporal pulse shaping. Here, it allows a chirping factor up to 85 (chirped pulses up to 8.5 ps) and the shaping process is performed on a spectral range of 25 nm providing a maximum of 110 channels corresponding to a maximum resolution of 0.23 nm. In the AOPDF crystal, the longitudinal interaction between the acoustic wave and the optical field polarized along the ordinary axis can be viewed as a three wave mixing process. This interaction results in the diffraction of optical wavelengths to the extraordinary axis depending on the phase-matching conditions. These conditions are given by:

1 $${{{\mathrm{k}}}}_{diff} = {{{\mathrm{k}}}}_{opt} + {{{\mathrm{k}}}}_{ac}$$

where k_*diff*_, k_*opt*_, and k_*ac*_ are the diffracted, optical and acoustic wavevectors, respectively. Depending on the angles of incidence of the acoustic wave and the optical field, the phase-matching conditions create an almost bijective relationship between the optical and acoustic frequencies (thick crystal). This relationship is nearly linear and mainly depends on the crystal properties in addition to the phase-matching conditions and is given by:

2 $$\omega_{opt}/\omega_{ac} \sim \alpha$$

where *α* is the optical to acoustic frequencies ratio. Here, *α* = 2.3 × 10^−7^. This enables pulse shaping by simply using an electronically generated acoustic wave. The shaping process in the AOPDF can be viewed as a convolution of the complex electric field of the input optical wave with the acoustic signal. In the frequency domain, this convolution can be expressed as:

3 $$E_{diff}(\omega_{opt}) = E_{in}(\omega_{opt}).S(\omega_{ac}) = E_{in}(\omega_{opt}).S(\alpha .\omega_{opt})$$

On the one hand, the amplitude of the diffracted electric field *E*_*diff*_ at a given angular frequency *ω*_*opt*_ is proportional to the input electric field *E*_*in*_ at the same angular frequency with a proportion determined by the amplitude of the acoustic wave with the frequency *α ω*_*opt*_ as dictated by the phase-matching conditions. The proper acoustic wave is obtained through the design of a mask while considering two main issues: spectral overlap removal and intensity equalization. In order to eliminate the spectral overlap between the sub-pulses, the mask is initially designed with the purpose of diffracting only non-overlapping spectral components. The sub-pulses are set to have a spectral width of 1.5 nm centred at *λ*_1_ = 788 nm, *λ*_2_ = 792 nm, *λ*_3_ = 802 nm, *λ*_4_ = 809 nm, and *λ*_5_ = 812 nm. After the elimination of the overlap, the spectra of the sub-pulses are proportional to the multiplication of the input spectrum with the AOPDF mask as stated by:

4 $${{{\mathrm{E}}}}_{{{{\mathrm{diff}}}}}\left( \lambda \right) \propto {{{\mathrm{E}}}}_{{{{\mathrm{in}}}}}\left( \lambda \right).\mathop {\sum}\limits_{i = 1}^5 {{{{\mathrm{U}}}}\left( {\lambda _{{{\mathrm{i}}}} - 0.75} \right) - {{{\mathrm{U}}}}\left( {\lambda _{{{\mathrm{i}}}} + 0.75} \right)}$$

Where *U* is the unit step function and *λ*_*i*_ is the central wavelength of sub-pulse *i*. As a result, the sub-pulses have a significant difference in their intensities depending on their position relative to the input spectrum. To fix this problem, the AOPDF mask is adjusted in the following manner:

5 $$\begin{array}{ll}{{{\mathrm{AOPDFmask}}}}\\ = {{{\mathrm{Norm}}}}\left( {\frac{1}{{{{{\mathrm{E}}}}_{{{{\mathrm{in}}}}}\left( \lambda \right).\mathop {\sum }\nolimits_{{{{\mathrm{i}}}} = 1}^5 {{{\mathrm{U}}}}\left( {\lambda _{{{\mathrm{i}}}} - 0.75} \right) - {{{\mathrm{U}}}}\left( {\lambda _{{{\mathrm{i}}}} + 0.75} \right)}}} \right)\end{array}$$

This way, the diffraction efficiency is adjusted for each sub-pulse to eliminate the intensity difference between them. The amplitude of the diffracted electric field can then be represented as:

6 $${{{\mathrm{E}}}}_{{{{\mathrm{diff}}}}}\left( \lambda \right) \propto \mathop{\sum} \limits_{{\mathrm{i}} = 1}^{5} {{{\mathrm{U}}}}\left( {\lambda _{{{\mathrm{i}}}} - 0.75} \right) - {{{\mathrm{U}}}}\left( {\lambda _{{{\mathrm{i}}}} + 0.75} \right)$$

As a result, we obtain five sub-pulses with the same spectral width and with quasi-equal intensities regardless of their spectral position with respect to the input spectrum.

On the other hand, the spectral phase of the diffracted optical pulse is simply the addition of the initial phase of the input optical pulse with the induced phase due to the acousto-optic interaction in the crystal.

7 $$\phi_{diff} = \phi_{opt} + \phi_{AOPDF}$$

*ϕ*_*AOPDF*_ is mere exploitation of the crystal birefringence to create a group delay between the wavelengths of the optical pulse depending on the position of the crystal at which they have been diffracted from the ordinary to the extraordinary axis. The group delay is given by:

8 $$\tau (\omega ) = (n_{go}(\omega )/c) \times z(\omega ) + (n_{ge}(\omega )/c) \times (L - z(\omega ))$$

With *n*_*go*_ and *n*_*ge*_ being the ordinary and extraordinary group refractive indices, respectively. c is the speed of light, *z*(*ω*) is the distance propagated by the optical frequency *ω* along the ordinary axis, and L is the total length of the crystal (L = 25 mm). By adjusting *z*(*ω*), the group delay of every optical frequency *ω* and the phase of the diffracted optical pulse can thereby be controlled. The phase induced by the acousto-optic interaction in the crystal is represented as a Taylor expansion up to the 4th order:

9 $$\phi _{{{{\mathrm{AOPDF}}}}} = \mathop{\sum} \limits_{{\mathrm{i}} = 1}^{4} - \left( {\frac{{{{{\mathrm{a}}}}_{{{\mathrm{i}}}}}}{{{{{\mathrm{i}}}}!}}\left( {\omega - \omega _0} \right)^{{{\mathrm{i}}}}} \right)$$

**Fig. 7:**
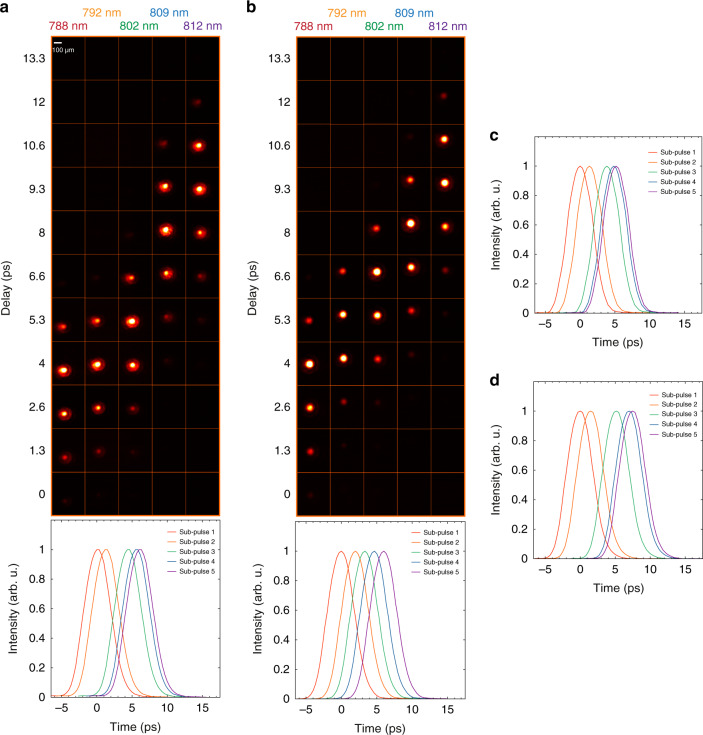
Temporal characterization of the system using OKG pump-probe experiments. **a** Images of the OKG as a function of the delay between the pump and the image pulse train for a_2_ = 40,000 fs^2^ without group delay adjustment (*top*) and the corresponding transmittance (bottom). **b** Images of the OKG as a function of the delay between the pump and the image pulse train for a_2_ = 40,000 fs^2^ with group delay adjustment (top) and the corresponding transmittance (bottom). **c** Transmittance of the OKG as a function of the delay between the pump and the image pulse train for a_2_ = 20,000 fs^2^. **d** Transmittance of the OKG as a function of the delay between the pump and the image pulse train for a_2_ = 60,000 fs^2^.

Where *ω*_0_ is the central frequency, *a*_1_ represents the group delay while *a*_2_, *a*_3_, and *a*_4_ represent the 2nd, 3rd, and 4th orders of the phase, respectively. Through the manipulation of acoustic wave’s phase, these coefficients can be adjusted in order to control the induced phase. The acoustic wave and the light pulse are then synchronized to interact within the acousto-optic crystal. The group delay is set so that the acoustic frequency phase-matched with the central wavelength of the input pulse is located at the center of the crystal at the moment of interaction. The coefficient *a*_2_ is set so that the acoustic wave is spread along the crystal to induce a linear variation of the instantaneous frequency of the input pulse, but also to increase the interaction length between the acoustic wave and the optical pulse, which allows to increase the diffraction efficiency of the AOPDF. For the 25 mm long crystal, the maximum value of coefficient *a*_2_ is around 60,000 fs^2^ for a bandwidth of 25 nm. At this value, the acoustic wave extends along the total length of the crystal and determines the total observation window of the SF-STAMP imaging system. In our experiment, the coefficient *a*_2_ is set to 20,000, 40,000, and 60,000 fs^2^ while searching for an optimal window of observation for the imaging system to capture the opening and closing of the optical Kerr gate (OKG) on a single shot. For each case, the transmittance of the OKG for the image pulses is measured in a pump-probe experiment as a function of the inter-pulse delay and the pump pulse. The image beam size was reduced (using a pinhole) to limit the spatial interaction with the pump beam in the CS_2_ cell. The transmittance of the OKG in time is a function of the delay between the pump and image pulses. Therefore, since the acousto-optic filtering results in the generation of five independent temporally separated pulses, the transmittance of the OKG for each of the five pulses is identical and is delayed in time with a delay corresponding to the time between the pulses (time between frames). This time can be easily obtained using cross-correlation. The transmittance of the OKG is shown in Fig. 7c, a and d for the three values of *a*_2_ in ascending order, while Fig. 7a shows the images of the OKG as a function of the delay between the pump and the image pulse train for *a*_2_ = 40,000 fs^2^. At this value, the AOPDF introduces an accumulated dispersion D = 0.269 ps/nm on a spectral range of ∆*λ*_*window*_ = 25 nm. This value was estimated using first-order linear regression as shown in Fig. 2e. The observation window ∆*T* of the imaging system in this case can be found using the following relation:

10 $${\Delta}T = D.{\Delta}\lambda _{{\mathrm{window}}}$$

For *a*_2_ = 40,000 fs^2^, the imaging system has a window of observation of 6 ps, which satisfies our need to observe the Kerr gate on a single shot, as demonstrated by numerical simulations. Using the accumulated dispersion along with the central wavelength and the spectral width of each sub-pulse, we are able to obtain the pulse width (exposure time, ET) of each of the five pulses using the following expression^26^:

11 $$ET\left( i \right) = \sqrt {\left( {\frac{{2\lambda _{0i}^2ln\,2}}{{\pi c{{{\mathrm{{\Delta}}}}}\lambda _i}}} \right)^2 + \left( {D.{{{\mathrm{{\Delta}}}}}\lambda _i} \right)^2}$$

where *i* is the pulse number, ∆*λ*_*i*_ is the spectral bandwidth and *λ*_0*i*_ is the central wavelength. The time between frames is further adjusted by tuning the group delay for 2nd, 3rd, and 4th pulses by modifying *a*_1_ for each of the concerned sub-pulses individually. Of course, this does not affect their pulse duration. The transmittance of the OKG for the image pulses is measured again as a function of the inter-pulse delay and the pump pulse to define the time separation between them. After a few iterations, the proper values of *a*_1_ are found and we obtained a quasi-constant time between frames as shown in Fig. 7b.

It is worth noting that the shaped pulse eventually exits the acousto-optic crystal at an angle of 1.4° with respect to the input pulse.

In the laser ablation experiment, the observation window was extended to the nanosecond time scale. In this case, the accumulated dispersion is the summation of the contributions of the modified single grating compressor and the AOPDF. Its value is estimated to be *D* = 1.01 ps/nm. Therefore, the average pulse duration amounts to *ET*_*average*_ ∼ 2 ps. As for the time between frames, it was precisely adjusted to 2.5 ns. Images of the laser ablation process were acquired on a small area using a microscope system comprising a f = 50 mm condenser lens and an objective lens (×20, NA = 0.35, Nachet). The beam was subsequently enlarged by a factor two using another telescope. The DOE and SF are eventually comprised within a 4 *f* imaging system with a magnification of one.

In the SF-STAMP detection, a diffractive optical element (DOE, Holoeye DE224) allows to generate 2 × 2 beams at a diffraction angle of 5.1° in addition to the non-diffracted beam. In order to select a different spectral band on each of the diffracted beams, a tilted band-pass spectral filter (BPF, IRIDIAN, ZX000167) is set after the DOE. It exhibits a transmittance superior to 90% at 800 nm and a spectral bandwidth ∆*λ*_*SF*_ of 2.2 nm. The BPF is adequately tilted in such a way to allow a selective transmission of each incident spectral component at a specific location on its surface. Here, the azimuthal and radial angles are found to optimal at *θ* ∼ 21° and *φ* ∼ 3° knowing that the central wavelength of the SF is angle-dependent and, for an angle *β* with respect to the normal of the SF plane, is given by^27,28^:

12 $$\lambda \left( \beta \right) = \lambda _0\left( {1 - \frac{{\beta ^2}}{{2n_{eff}}}} \right)$$

From this, the corresponding wavelength-dependant transmitted intensity can be expressed as:

13 $${{{\mathrm{I}}}}\left( \lambda \right) = {{{\mathrm{exp}}}}\left( {\frac{{ - 4{{{\mathrm{ln}}}}\left( 2 \right)\left( {\lambda - \lambda \left( \beta \right)} \right)^2}}{{{{{\mathrm{{\Delta}}}}}\lambda _{SF}^2}}} \right)$$

Finally, the five sub-pulses are eventually captured using a standard CCD camera (JAI, RM-4200 CL) with 2048 × 2048 pixels and 15.15 × 15.15 mm sensor dimensions.

### OKG imaging

In our experiment, the femtosecond laser pulse was linearly polarized and split by a 90/10 beam splitter prior to the pulse-shaping stage as shown in Fig. 3a. Ninety percent of the power was used as a pump while the rest was directly injected into the AOPDF. At the output of the AOPDF, we obtained five linearly polarized pulses with equal intensity and temporal and spectral separations as discussed in the previous section. A CS_2_ cell and a single polarizer were eventually set between the AOPDF and the lensless SF-STAMP as a Kerr time-gate. The pump pulses were synchronized with the image pulses via an optical delay line and a cylindrical lens (f = 75 mm) was used in order to focus the pump beam on a single axis (vertical) inside the 10 × 10 mm CS_2_ cell at an angle of 42° with respect to the image beam.

### A theoretical model for the optical Kerr gate

In the simulation of the CS_2_-based OKG, we considered the change of the refractive index in time and space due to the pump intensity as follows^42^:

14 $$\begin{array}{lll}{{{\mathrm{{\Delta}}}}}n\left( {{{{\mathbf{r}}}},t} \right) = n_2\frac{{\tau _0 + \tau _r}}{\tau }\mathop{\int} \limits_{ - \infty }^{t} I_p\left( {r,t} \right)exp\\\left[ { - \frac{{t - \tau }}{{\tau _0}}} \right]\left[ {1 - exp\left[ { - \frac{{t - \tau }}{{\tau _r}}} \right]} \right]d\tau\end{array}$$

with *t* ≥ 0, where *τ*_0_ is the rise time (*τ*_0_ = 0.14 ps), *τ*_*r*_ the relaxation time (*τ*_*r*_ = 1.0 ps), *n*_2_ the nonlinear refractive index of CS_2_ (*n*_2_ = 3.0 × 10^−19^*m*^2^*/W*)^42^ and *I*_*p*_(r,*τ*) the intensity of the pump in time and space. The pump beam is given an elliptical spatial profile and a Gaussian temporal shape corresponding to an intensity distribution given by:

15 $$I_p\left( {{{{\boldsymbol{r}}}},t} \right) = I_{pmax}exp\left[ { - \frac{{x^2}}{{x_p^2}} - \frac{{y^2}}{{y_p^2}} - \left( {\frac{t}{{\tau _p}}} \right)^2} \right]$$

with *x*_2_ = 3.8 mm (corresponding to a FWHM = 5.3 mm), *y*_2_ = 1.4 mm, *τ*_*p*_ = 60 *fs* (FWHM = 100 fs). The energy per pulse is E = 0.4 mJ and *I*_*pmax*_ (in W/m^2^) is the maximum intensity of the pump. The image is composed of 5 pulses delayed in time, which all have the same Gaussian shape, in space and time. Nevertheless, as there is no overlap between the pulses on the detector, only one pulse propagation is simulated, and is delayed by a time *τ*_*d*_ to mimic the other pulses. The probe pulse is thus written as:

16 $$I_{im}\left( {{{{\boldsymbol{r}}}},t} \right) = I_{immax}exp\left[ { - \frac{{x^2 + y^2}}{{r_{im}^2}} - \left( {\frac{{t - t_d}}{{\tau _{im}}}} \right)^2} \right]$$

where *r*_*im*_ = 1.0 mm (FWHM = 1.6 mm), *τ*_*im*_ = 0.41 ps (FWHM = 0.723 ps), *τ*_*d*_ = 0.0, 2.0, 3.3, 4.7, 6.0 ps and *I*_*immax*_ is the maximum intensity of the image pulses. The CS_2_ cell is rectangular and much larger than the overlap zone of pump and probe pulses. The length of the cell in the image beam propagation direction *z* is 10 mm. The angle between the two beams is Θ = 42° in air, corresponding to 26° inside the cell according to the Snell-Descartes laws. The pump beam induces a birefringence at an angle of 45° relative to the *x*- and *y*-axes. The phase change of the image pulse along a propagation distance *δz*, where image and pump pulses overlap, is given by:

17 $$\delta \varphi = \frac{{2\pi \delta z}}{\lambda }{{{\mathrm{{\Delta}}}}}n$$

For each time interval, the overlap between the image and pump pulses is computed and the phase change *δφ* is deduced. The overall transmitted intensity is finally given by Eq. 18, where *φ* is the accumulation of all *δφ*.

18 $$I = {\mathrm{sin}}^2\frac{\varphi }{2}$$

### Digital in-line holography (DIH)

The recording process of digital in-line holograms can be simply expressed by applying a propagation model along the z-axis (i.e., the optical axis of the imaging system). Let *A*_*im*_(r,*t*) be the complex amplitude function due to the probe pulse *I*_*im*_(r,*t*) and considered as the object to be recorded. The complex amplitude distribution in the CCD sensor plane located at a distance *z*_*e*_ from the object is obtained from the Huygens-Fresnel integral. According to ref. ^47^ and by omitting the constant multiplicative phase term ${\mathrm{exp}}\left( {\frac{{2i{\uppi}z_e}}{\lambda }} \right)$, this integral can usefully be expressed as the following 2-D convolution:

19 $$A_{{\mathrm{holo}}}(r,t,z_e) = [1 - A_{im}(r,t)] \ast \ast h(r,z_e)$$

where *h*(r,*z*_*e*_) is the Fresnel kernel:

20 $$h\left( {{{{\boldsymbol{r}}}},z_e} \right) = \frac{1}{{i\lambda z_e}}{\mathrm{exp}}\left[ {\frac{{i{\uppi}\left( {x^2 + y^2} \right)}}{{\lambda z_e}}} \right]$$

The intensity distribution $\left( { = A_{{\mathrm{holo}}}.\overline {A_{{\mathrm{holo}}}} } \right)$ recorded by the sensor can also be expressed by using this formalism.

Knowing that 1 ∗ ∗*h* = 1, it gives:

21 $$I_{{\mathrm{holo}}}\left( {{{{\boldsymbol{r}}}},t,z_e} \right) = 1 - A_{im}\left( {{{{\boldsymbol{r}}}},t} \right) \ast \ast \left[ {h\left( {{{{\boldsymbol{r}}}},z_e} \right) + \overline {h\left( {{{{\boldsymbol{r}}}},z_e} \right)} } \right]$$

where the top bar ¯. stands for the complex conjugate.

Note here that for simplification, the squared modulus term |*A*_*im*_(r,*t*)∗∗*h*(r,*z*_*e*_)|^2^ has been omitted. This approximation is valid provided that the far-field conditions are satisfied^48^.

As for the recording step, the reconstructed image at a given distance *z*_*r*_ from the CCD sensor can also be calculated by a convolution operation:

22 $$R(r,t,z_r) = I_{{\mathrm{holo}}}(r,t,z_e) \ast \ast h(r,z_r)$$

It is easy to see, by introducing Eq. 21 in 22, that when the right reconstruction distance is reached (i.e., *z*_*r*_ = *z*_*e*_ = *z*_*opt*_), we have:

23 $$R(r,t) = 1 - A_{im}(r,t) - A_{im}(r,t) \ast \ast h(r,2z_{opt})$$

The reconstructed complex image amplitude 1 − *A*_*im*_(r,*t*) is surrounded by the waves coming from the so-called twin image −*A*_*im*_(r,*t*) ∗ ∗*h*(r,2*z*_*opt*_). In the present case, the far-field conditions are achieved and we assume that the contrast of the twin image fringes does not disturb the reconstructed sample volume. Examples of hologram recording (see Fig. 6a) and reconstruction (see Fig. 6b) are presented. The phase values of *R*(r,*t*)—here mapped in the (*y*,*z*) plane—are shown in Fig. 6c. It is noticeable that this orthogonal phase map representation is quite similar to the phase signature presented in ref. ^45^. The phase discontinuity observed here allows efficient detection of the best focusing plane in the reconstruction volume. In the example shown in Fig. 6c, the object axial coordinate has been estimated to *z*_*opt*_ = 539 mm.
